# Embossing-Controlled Hierarchical Structures in Cellulose-Derived Carbon Paper for Flexible Piezoresistive Pressure Sensing

**DOI:** 10.3390/molecules31203736

**Published:** 2026-10-18

**Authors:** Wei Wang, Luyao Chen, Zhixiang Wang, Youbo Di, Yaru Yang, Yunchao Xiao, Shuo Yang, Hua Wang

**Affiliations:** 1 College of Textile Engineering, Taiyuan University of Technology, Taiyuan 030600, China; 2 College of Materials and Textile Engineering, Jiaxing University, Jiaxing 314001, China; 3 School of Light Industry Science and Engineering, Tianjin University of Science and Technology, Tianjin 300457, China; 4 State Key Laboratory of Bio-Based Fiber Materials, China Textile Academy, Beijing 100025, China

**Keywords:** embossing, flexible pressure sensor, patterned array, micro-bump structures, carbon paper

## Abstract

Engineering three-dimensional (3D) microstructures in sensing layers is an effective strategy for improving the performance of flexible pressure sensors. However, most technologies for constructing 3D microstructures involve complex fabrication procedures and high costs, which hinder the commercialization of flexible electronics. Herein, inspired by embossing in papermaking, we developed a flexible piezoresistive pressure sensor based on carbonized embossed paper (CEP). Embossing followed by carbonization produced a hierarchical structure comprising a porous fibrous network and an array of surface micro-bumps. The spatial frequency and geometry of the micro-bump array were adjusted using metal mesh molds with different mesh counts. The CEP-50 sensor exhibited a sensitivity of 70.14 kPa^−1^ over 0–0.2 kPa, a working range of 0–150 kPa, a response time of approximately 60 ms, and stable responses over 5000 loading–unloading cycles. The CEP-50 sensor exhibited a sensitivity of 70.14 kPa^−1^ over 0–0.2 kPa, a working range of 0–150 kPa, a response time of approximately 60 ms, a low detection limit of 1.5 Pa, and stable responses over 5000 loading–unloading cycles. An equivalent circuit and finite element analysis were used to develop a qualitative interpretation of the pressure-dependent electrical response. The sensor was further demonstrated for monitoring pulse, respiration, phonation, and body movements. This embossing-based fabrication approach provides a practical route for developing paper-based wearable pressure sensors.

## 1. Introduction

With the rapid development of the Internet of Things, flexible wearable devices are receiving widespread attention due to their considerable application prospects in vast areas, such as soft robotics [1,2,3], medical monitoring [4,5,6], body-motion detection [7,8,9], and human–machine interactions [10,11,12]. As a vital element of flexible wearable devices, flexible pressure sensors can convert external pressure signals into electrical signals [13,14]. Specifically, based on different electromechanical behaviors, flexible pressure sensors can measure pressure signals through piezoresistive, capacitive, piezoelectric and triboelectric effects [15,16,17]. Among these devices, piezoresistive pressure sensors are widely investigated because of their relatively simple structures and straightforward electrical readout [18,19]. Typically, a flexible piezoresistive pressure sensor is made up of sensing materials, electrodes, and flexible substrates. Pressure-induced changes in the conductive network generate the electrical response, while the substrates provide mechanical support and flexibility [20,21]. Therefore, the composition and structure of the sensing layer play important roles in determining sensor performance.

The choice of conductive materials and the structural design of the sensing layer are key factors governing sensor performance [22,23,24]. Common conductive materials include carbon nanotubes (CNTs), reduced graphene oxide (rGO), MXene, silver nanowires (AgNWs), and copper nanowires (CuNWs) [25,26,27]. However, these materials are expensive and difficult to prepare, which is a major challenge to propel the industrialization of flexible wearable sensors. In addition, the use of geometric microengineering techniques to design the structures of the sensing layer, such as micro-domes [28], micropyramids [29], microspheres [30], micropores [31], microcylinders [32] and porous structures [33], has emerged as a desirable approach for improving the sensing performance of flexible sensors. For example, Gao et al. presented a typical wearable pressure sensor with micro-bump structures using laser microengineering techniques, including micro-domes and micro-ridges. By combining finite element simulation, they found that the micro-dome is more helpful for enhancing the sensing sensitivity of the pressure sensor compared to other types of micro-bump structures, which was about 17 times (GF of −1.82 kPa^−1^) more sensitive than that of other sensors [34]. Nevertheless, the common methods of geometric microengineering, including photolithography [35], etching [36], three-dimensional (3D) printing [37] and mold replication [38,39], are complicated, costly, and time-consuming, which may hinder the commercialization of these flexible pressure sensors. Thus, fabricating high-performance flexible pressure sensors via cost-effective and large-scale methods still presents a great challenge.

Paper is an attractive substrate for flexible sensors because of its low cost, flexibility, and porous fibrous structure. Its interconnected fiber network can support conductive materials and provide a deformable framework for pressure sensing [16,40]. Recent studies have demonstrated several approaches to constructing conductive sensing networks on paper substrates. Wei et al. [41] prepared MXene/filter-paper pressure sensors via impregnation and achieved a broad pressure detection range extending to 640 kPa. Chen et al. [42] combined a friction-coated water-soluble graphite-sensing layer with screen-printed carbon interdigital electrodes, demonstrating that simple paper-based fabrication methods can deliver high sensitivity and a wide detection range. Li et al. [43] investigated cellulose-based paper substrates with different fiber microstructures and incorporated MXene by dip-coating, highlighting the influence of the fibrous substrate on sensing performance. Zhao et al. [33] proposed an all-paper, cuttable, and foldable pressure sensor assembled with two paper-based materials with different conductivities. Gao et al. [5] fabricated an all-paper-based pressure sensor by assembling tissue paper loaded with AgNWs and nanocellulose paper printed with interdigitated electrodes. The obtained paper-based sensor has a sensitivity of 1.5 kPa^−1^ in the working range of 0.03–30.2 kPa. These advances demonstrate that paper-based pressure sensors can achieve high sensitivity and broad detection ranges through conductive functionalization and structural design. They also highlight the importance of examining how the fabrication route and the resulting architecture influence pressure-dependent electrical responses.

Hierarchical structures can tailor the pressure response of flexible piezoresistive sensors by combining deformation within a porous network with changes in interfacial contact [28]. In our recently published work [6], we proposed an all-fiber-based flexible pressure sensor with multi-level microstructures, demonstrating the synergistic optimization of microstructure and porous structure on the sensitivity, detection limit, and working range of pressure sensors. Lai et al. [44] used pencil graphite frottage over a sandpaper template to produce graphite-coated paper with embossed microstructures. These studies motivate further investigation of fabrication routes that combine surface patterns with porous sensing networks. Embossing is an established papermaking technique for producing three-dimensional surface patterns. In the present study, metal mesh embossing was applied before carbonization to generate patterned carbon paper, and the relationship between the resulting structure and sensing performance was examined [42].

In this work, inspired by embossing patterns, we demonstrated a facile and effective strategy for constructing the surface micro-bump pattern structure of flexible paper-based pressure sensors via embossing. The metal mesh was chosen as the embossed mold to give the pristine paper embossed patterns using simple hot pressing. Subsequently, the embossed paper with micro-bump patterns after carbonization was used as sensing material in flexible sensors. Benefiting from the combination of a 3D fibrous network porous structure inside the paper and a micro-bump pattern structure on the paper surface, the obtained paper-based pressure sensor exhibited superior sensing performance, such as ultrahigh sensitivity (70.14 kPa^−1^), low detection limit (1.5 Pa), and a wide working range (0–150 kPa). The effect of the patterned array microstructure on the sensing mechanism can be unveiled through finite element analysis and an equivalent circuit. Additionally, the as-fabricated paper-based flexible sensor can serve as a monitor to detect various body movements, including physiological or motion signals. This facile, cost-effective, scalable, and sustainable design strategy and preparation technique offers a novel direction for the commercialization of flexible electronics.

## 2. Experimental Section

### 2.1. Materials

Commercial cellulose air filter paper (model LW16/54CW) was purchased from Shandong Longde Composite Fiber Technology Co., Ltd. (Weifang, China). The paper had a basis weight of 120 g m^−2^, a thickness of 280 μm, an air permeability of 450 ± 50 L m^−2^ s^−1^, a stiffness of >4.0 mN·m, and a maximum pore size of 80 μm. The metal mesh, silver paste, copper wires, and polyimide film tape were purchased from Tianjin Guangfu Technology Co., Ltd. (Tianjin, China).

### 2.2. Fabrication of CEP Pressure Sensors

**Figure 1:**
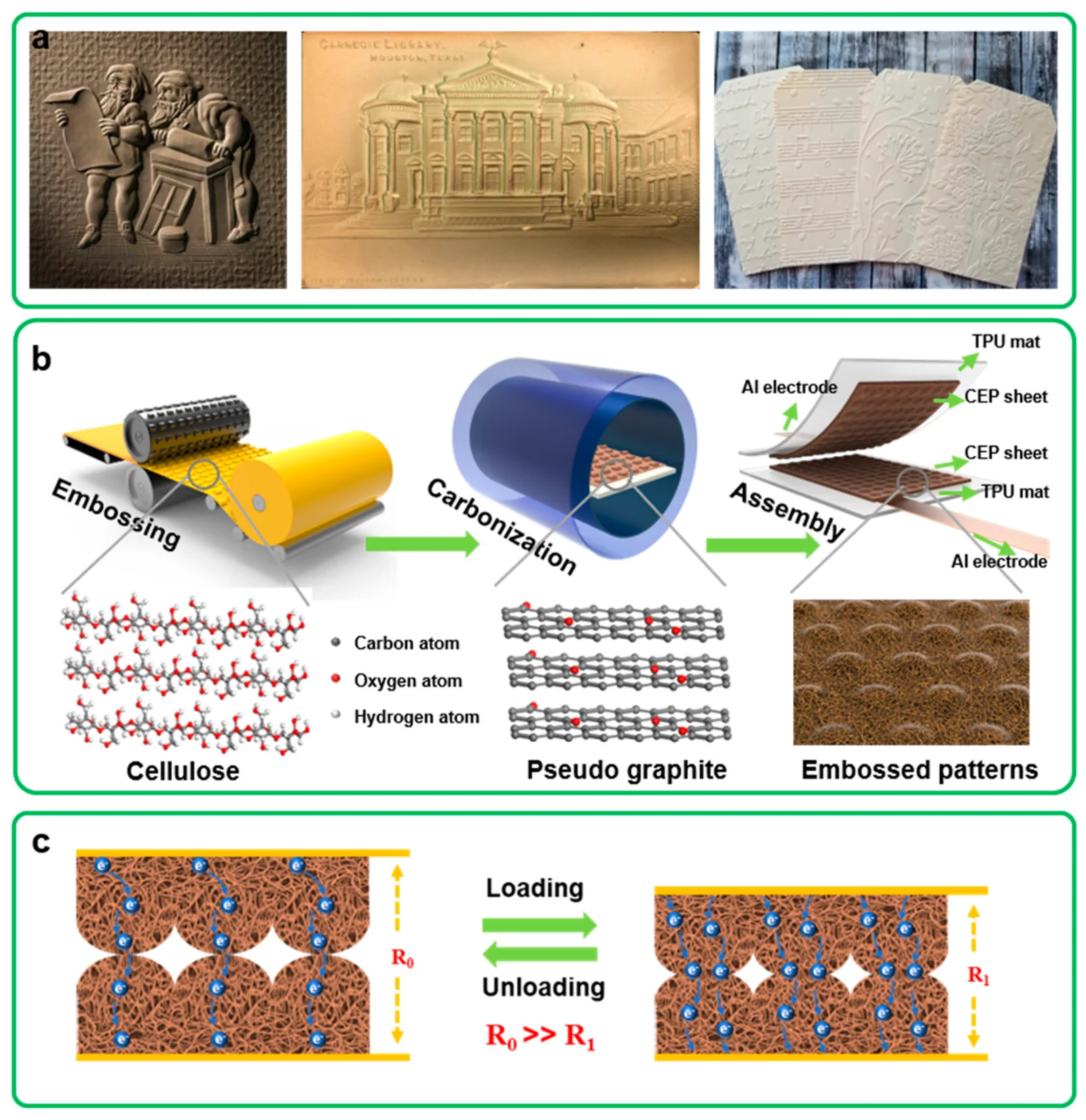
Illustration of design concept and assembly process of flexible CEP pressure sensor. (**a**) Some common patterns of embossed paper in daily life. (**b**) Fabrication of flexible CEP pressure sensor. (**c**) Sensing mechanism of flexible CEP pressure sensor, where contact deformation provides more channels for electron migration, thus making total resistance lower.

Commercial cellulose air filter paper with a basis weight of 120 g/m^2^ was utilized as a raw material. Embossed paper was prepared using various metal meshes (30, 50, 70, 100) as embossing molds. The metal mesh was placed on air filter paper and was hot-pressed at 105 °C for 10 min using a pressure of 4 MPa. The obtained embossed paper was carbonized in a tube furnace with the protection of a nitrogen atmosphere according to the following temperature control procedure: (1) heated from 20 to 200 °C with a heating rate of 20 °C min^−1^ held for 1 h; (2) heated to 400 °C with a heating rate of 10 °C min^−1^ held for 2 h; (3) then heated to 950 °C with a heating rate of 5 °C min^−1^ held for 2 h; and (4) natural cooling to 30 °C. The carbonized embossed paper (CEP) was trimmed into a square with a width of 15 mm and served as the sensing layer of the flexible pressure sensor. The assembly procedure of the CEP pressure sensor is illustrated in Figure 1b. A CEP sheet (the size of 15 mm × 15 mm) was transferred onto an electrospun TPU fibrous mat (with a thickness of 100 μm) with the aluminum foil electrodes (with a thickness of 60 μm) as electrodes. A TPU fibrous mat was fabricated by electrospinning and used as a breathable and flexible substrate [6]. The specific spinning parameters are described in our previous work. Lastly, two CEP sheets, electrodes and electrospun TPU fibrous mats were assembled to fabricate the CEP pressure sensor. The detailed layer arrangement and device architecture are illustrated in Figure 1b.

### 2.3. Characterization and Electromechanical Measurements

Sample morphologies were examined using field-emission scanning electron microscopy (FE-SEM, FEI Quanta 650) (FEI Company, Hillsboro, OR, USA). Raman spectra were characterized using a laser excitation wavelength of 532 nm (LabRAM HR Evolution) (HORIBA France SAS, Palaiseau, France). The 3D surface structures of carbonized embossed paper were observed using a scanning probe microscope (SPM) (FPUNI-usm1400) (UNISOKU, Hirakata, Japan). The surface roughness of embossed paper was measured by the Parker Print-Surf (PPS) method (TMI M590) (TMI, New Castle, DE, USA). The electromechanical performance of the CEP sensors was evaluated using a custom-built testing platform comprising a universal material testing machine (TSE504C) (WANCE, Shenzhen, China) and a Keithley source meter (Model 2450) (Keithley Instruments, Solon, OH, USA). The CEP sensor was placed on the universal material testing machine, and the mechanical loading protocol, including cyclic loading–unloading and loading frequency, was controlled using a computer. The two aluminum-foil electrodes were connected to a Keithley source meter in a two-wire configuration, and the current response was recorded at a constant bias voltage of 2 V. The current–voltage (I–V) curves were obtained by sweeping the voltage from −0.1 to 0.1 V in steps of 0.002 V while maintaining a fixed applied pressure. To monitor human motion signals, the sensors were attached to the corresponding body locations, and the electrical responses were recorded in real time.

### 2.4. Finite Element Analysis Approach

Finite element analysis was performed using ABAQUS/CAE 6.14 and the Abaqus/Standard solver to investigate the mechanical response of the embossed CEP structure under compression. A simplified three-dimensional model comprising two identical CEP layers was constructed, with the micro-bump dimensions determined from SEM images of the CEP sheets. Each layer was represented as an equivalent continuum and discretized using eight-node reduced-integration brick elements (C3D8R). Orthotropic elasticity was defined using Young’s moduli of E_1_ = 790, E_2_ = 504.34, E_3_ = 2.52, G_12_ = 244.34, and G_13_ = G_23_ = 22.57 MPa; shear moduli of G_12_ = 244.34, and G_13_ = G_23_ = 22.57 MPa; and Poisson’s ratios of v_12_ = 0.34, and v_13_ = v_23_ = 0.01. Hill anisotropic plasticity was specified using a reference yield stress of 48.53 MPa at zero plastic strain and yield-stress ratios of R_11_ = 1, R_22_ = R_33_ = 0.5, and R_12_ = R_13_ = R_23_ = 0.12. Only one yield-stress–plastic-strain data point was specified; no additional strain-hardening data were provided. General contact was defined over the exterior surfaces, with hard contact in the normal direction and a friction coefficient of 0.2 in the tangential direction. A designated node set on the first CEP layer was fixed against translation. The loading node set on the second layer was constrained in the first and second directions, while a displacement of −0.002 mm was prescribed in the third direction. The displacement was applied using a linear ramp over a static step with a step-time parameter of 1. Geometric nonlinearity was disabled. Automatic incrementation was used, with initial, minimum, and maximum increments of 0.05, 10^−5^, and 0.05, respectively. The simulations were used to examine deformation and stress distributions and provide qualitative mechanical support for the proposed sensing interpretation. Electrical transport was not included; therefore, the model did not quantitatively predict sensitivity or independently determine the contributions of contact and volume resistance.

## 3. Results and Discussion

### 3.1. Concept of Paper-Based Flexible Pressure Sensors with Patterned Array Microstructures and Their Structural Characterizations

Embossed paper is a delicate art paper with three-dimensional patterns that is widely used for elegant book covers, high-end packaging, and luxury decoration. As illustrated in Figure 1a, these images are examples of embossed paper that are common in our lives. Inspired by the embossing pattern, we proposed a facile, effective fabrication technology for a high-performance flexible pressure sensor, including embossing, carbonization and assembly. The assembly process of the flexible CEP pressure sensor is shown in Figure 1b: emboss pristine cellulose paper to form a uniform array of micro-bump patterns on one side of the paper; carbonize the embossed paper under an inert atmosphere to drive the conversion of the pseudo-graphite structure from cellulose; and assemble two sheets of carbonized embossed paper in a face-to-face format to create a CEP-based flexible pressure sensor. After embossing and carbonization, the conductive paper-based sensing layer with multi-level microstructures was obtained, consisting of a 3D fibrous network porous structure inside the paper and a micro-bump pattern structure on the paper surface. The advantages of the above structure and processes are as follows:

(1) The paper materials used in this study include cheap and easy-to-obtain air filter paper with high formation and bulk, which ensures the compressibility and stability of the paper-based sensing layer.
(2) The micro-bump pattern structure on the surface of the paper causes a rapid change in resistance due to the stress concentration effect when pressure is applied (as shown in Figure 1c), resulting in high sensitivity (especially under low-pressure conditions).
(3) The fibrous network porous structure inside the paper improves the working range of the sensor.
(4) The embossing technique is a well-established, efficient, and low-cost process in papermaking, and its implementation is simple and straightforward, making it possible to mass-produce flexible pressure sensors.

**Figure 2:**
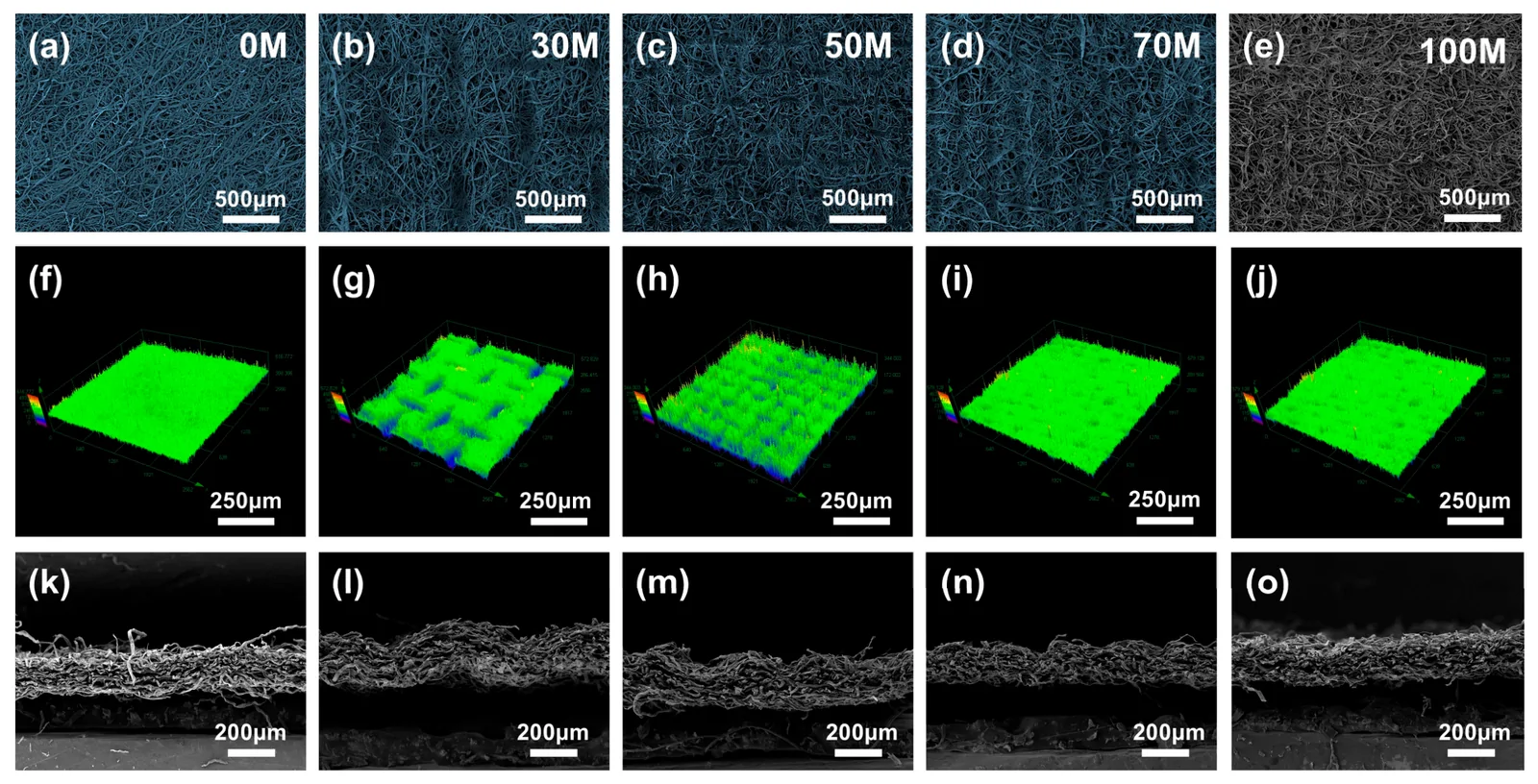
Morphology of carbonized embossed paper with different metal molds. (**a**–**e**) SEM images of carbonized embossed paper with different metal molds (0, 30, 50, 70, 100 mesh numbers). (**f**–**j**) SPM images of carbonized embossed paper with different metal molds (0, 30, 50, 70, 100 mesh numbers). (**k**–**o**) The cross-section of carbonized embossed paper with different metal molds (0, 30, 50, 70, 100 mesh numbers).

In this work, the mold pattern used for embossing is formed using metal meshes with different mesh numbers (30, 50, 70, and 100), which are woven with metal wires of different diameters to form various mesh densities (refer to Figure S1). Figure S2 presents the dimensions of the embossed paper before and after carbonization. It is clear that after carbonization, its dimensions shrink, and its length and width are reduced by 20% and 18%, respectively. Intriguingly, its pattern is still well preserved, as depicted in SEM images (Figure 2a,b). As seen in Figure S3, X-ray photoelectron spectroscopy (XPS) demonstrates the changes in the main elements of embossed paper before and after carbonization [45]. Before carbonization, the C 1s spectrum is dominated by the C–O component, with additional contributions from C–C and O–C–O. After carbonization, C–C becomes the predominant component, whereas the C–O contribution decreases and O–C–O is no longer separately resolved. This change is consistent with the removal of oxygen-containing groups during carbonization. The Raman spectra of embossed paper before and after carbonization are shown in Figure S5. The band at 1096 cm^−1^ is typically attributed to overlapping C–O and C–C stretching vibrations, and the band at 2895 cm^−1^ is associated with C–H stretching vibrations of the CH and CH_2_ groups in cellulose [46]. After carbonization, 1353 and 1594 cm^−1^ represent the D-band and G-band [47], respectively, where the G-band is associated with crystalline sp2, while the D-band is related to defects or heteroatom doping. The peak intensity ratio (I_D_/I_G_) of the defective (D band) and crystalline (G band) carbon is 0.9, implying that carbonized embossed paper has a partially graphitized structure.

**Figure 3:**
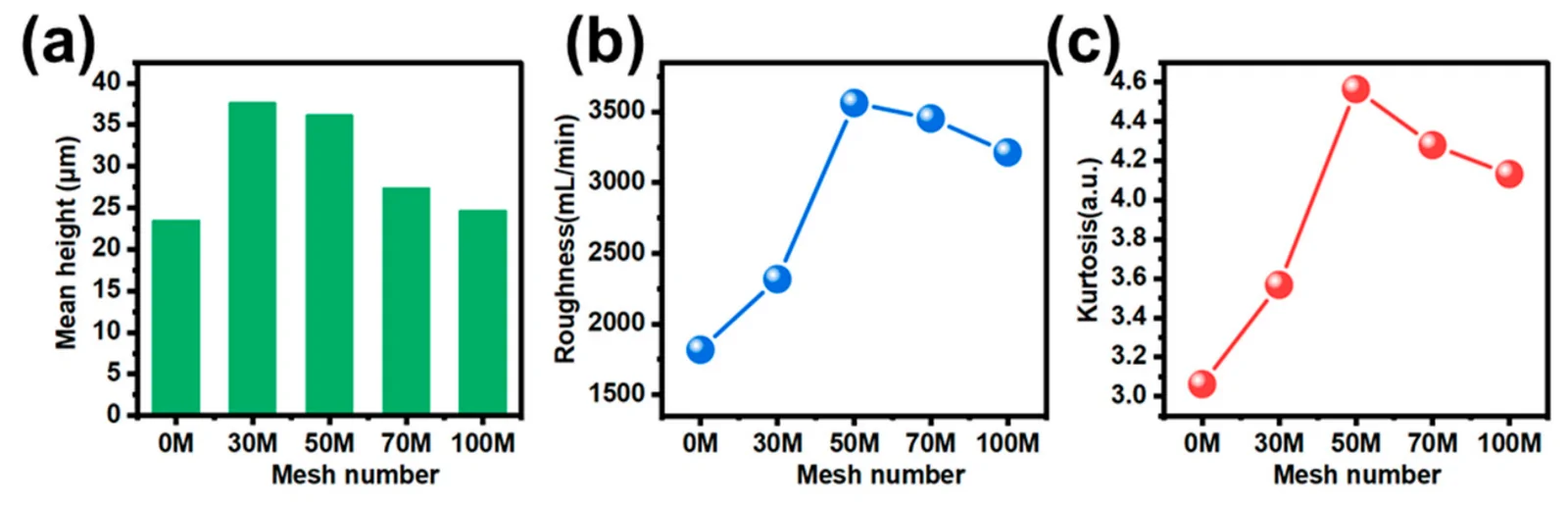
The roughness of the embossed paper and the geometric parameters of the embossed pattern. (**a**) The mean height of the embossed pattern versus the mesh number of the metal mold. (**b**) The roughness of the embossed paper versus the mesh number of the metal mold. (**c**) The kurtosis of the embossed pattern versus the mesh number of the metal mold.

The surface pattern of embossed paper is closely related to the selected embossing mold. As shown in Figure 2b, after embossing with a 30 M mold, its surface becomes a uniform array of micro-bump patterns from a flat pattern. Meanwhile, the size, density, and geometry of these surface patterns can be adjusted using molds with different mesh numbers. Figure 2b–e show SEM images of CEP-30, CEP-50, CEP-70, and CEP-100. It is obvious that the spatial frequency of the embossed pattern correlates with the mesh number of the metal mold: a larger mesh number produces more closely spaced micro-bumps with smaller lateral dimensions. Furthermore, in order to more finely characterize the microstructures after embossing, the scanning probe microscope (SPM) was used to confirm the geometries of these patterns (Figure 2f–j). In Figure 2k–o, the cross-section images of carbonized pristine paper (CPP), CEP-30, CEP-50, CEP-70, and CEP-100 are shown. Meanwhile, according to the height of the embossed pattern measured by the SPM, the effects of mesh number on the height of the embossed pattern were further analyzed. As shown in Figure 3a, the height of the embossing patterns decreases with increasing mesh count for the used embossing molds. This decrease is attributed to the combined effects of reduced wire crimp depth and smaller mesh apertures, which restrict the penetration of cellulose fibers into the mesh openings during pressing.

According to recently published studies [42,43,48,49], a rough sensing layer is beneficial to improve the sensitivity of the pressure sensor due to the fact that rough surface structures can produce greater structural deformation when subjected to pressure. Figure 3b shows the effect of the embossing pattern on its surface roughness: CEP-50 has the largest roughness with 3562.6 mL/min, which is about two times that of the pristine paper. Furthermore, the kurtosis of the embossing pattern is measured, which is often used to indicate the sharpness of a graphic. In Figure 3c, CEP-50 shows the largest kurtosis, indicating that its micro-pattern is sharper compared to the others. As a sensing layer, this sharp surface structure has two distinct advantages over a flat surface structure: (1) the initial contact area between the sensing layers is reduced, thus greatly increasing its initial resistance, and (2) the deformation is greater when subjected to pressure, causing a more pronounced change in contact resistance.

### 3.2. Sensing Performance

**Figure 4:**
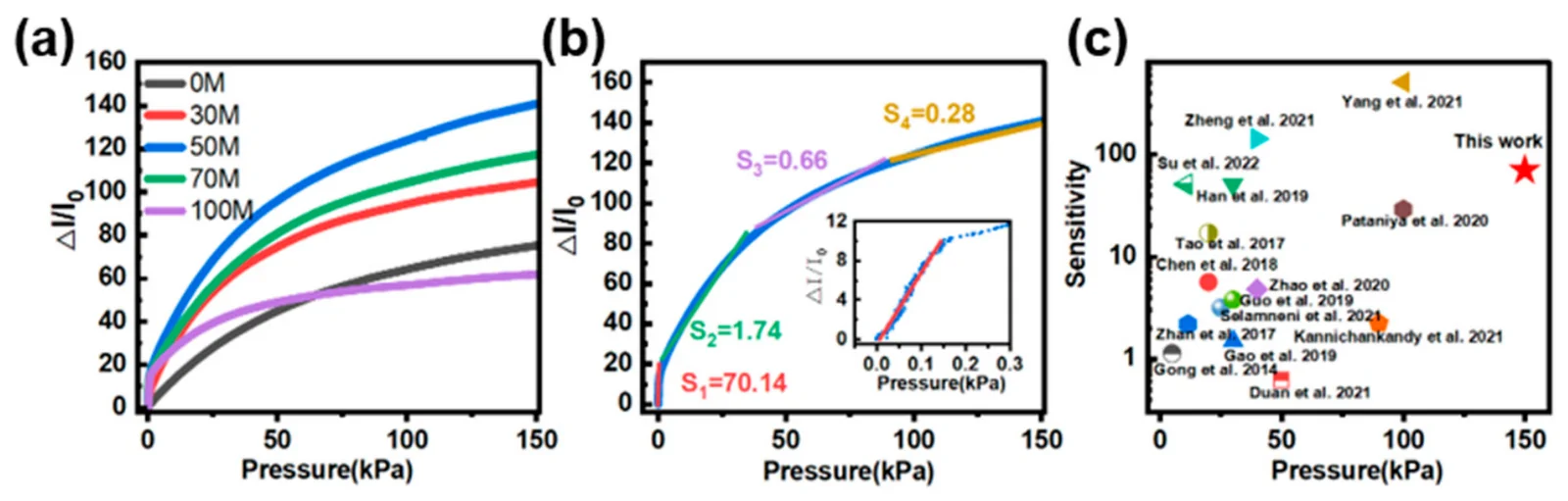
The effect of the metal mold’s mesh number on the sensitivity of the flexible pressure sensor. (**a**) Relative current variation in flexible CEP sensors embossed by various metal molds (0, 30, 50, 70, 100 mesh numbers). (**b**) Division of linear sensitivity for flexible CEP-50 pressure sensors (the sensitivity from 0 to 0.3 kPa is shown in the inset image). (**c**) Performance comparison of this work with recently reported flexible paper-based pressure sensors [1,5,11,14,29,32,33,35,39,45,46,47,50,51,52].

The sensing performance of the flexible CEP pressure sensor is shown in Figure 4. The structure parameters of embossed paper have a significant influence on its sensing performance. Sensitivity refers to one of the few very significant capability parameters for flexible sensors, representing the relationship between the relative current change slope and the corresponding pressure [50]. Typically, sensitivity is defined as S = {(I − I_0_)/I_0_}/ΔP, where I and I_0_ denote the real current and initial current, and ΔP is the corresponding pressure [51]. In Figure 4a, the relative current change in these flexible CEP sensors is different under the same pressure, mostly due to the structural variability of their embossed patterns. While subjected to pressure, the relative current change in the flexible CEP-50 pressure sensor becomes most obvious, indicating that it has the highest sensitivity.

In Figure 4b, the sensitivity of the flexible CEP-50 pressure sensor is divided into four linear regions: 70.14 kPa^−1^ (S_1_) in the 0–0.2 kPa pressure range, 1.74 kPa^−1^ (S_2_) in the 0.2–35 kPa pressure range, 0.66 kPa^−1^ (S_3_) in the 35–90 kPa pressure range, and 0.28 kPa^−1^ (S_4_) in the 90–150 kPa pressure range. Compared to the flexible pressure sensor with pristine paper, its maximum sensitivity was increased 33 times. Figure 4c illustrates the comparison of the flexible CEP-50 pressure sensor with paper-based flexible pressure sensors referred to in the literature in terms of their sensitivity and sensing range, and a more thorough capability comparison is presented in Table S1. The differences in pressure response among the CEP sensors indicate that the embossed surface structure influences the overall electromechanical behavior. For the different embossing molds, changes in mesh count altered the micro-bump height, lateral dimensions, spacing, and surface characteristics. In the face-to-face configuration, micro-bump geometry and spatial arrangement may influence the initial contact between the CEP sheets and their evolution during compression, thereby affecting the resistance response. Although CEP-50 did not exhibit the greatest micro-bump height, it showed the highest surface roughness and kurtosis (Figure 3a–c). These results indicate that the surface roughness of the micro-protrusions contributes more to improving sensing sensitivity than their height.

**Figure 5:**
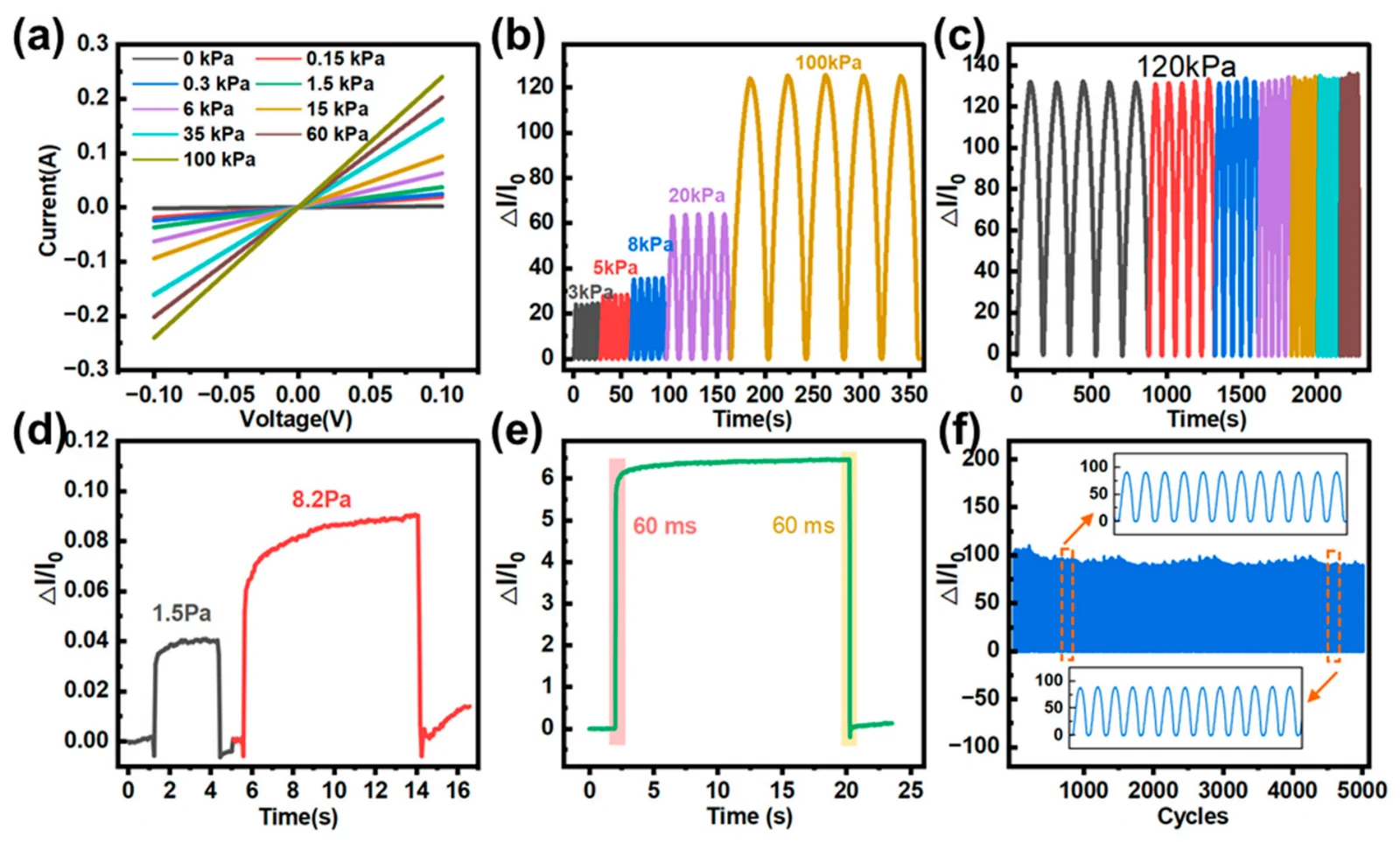
Electromechanical features of the flexible CEP sensor. (**a**) I–V curves of the CEP sensor under various pressures (applied from 0 to 100 kPa). (**b**) Relative current variations in the CEP sensor under various pressures at a constant frequency of 0.01 Hz (from 3 to 100 kPa). (**c**) Relative current changes in the CEP sensor under pressure of 120 kPa at disparate frequencies (from 0.005 to 0.04 Hz). (**d**) Relative current variations in the CEP sensor under subtle pressure (around 1/8 and 1 silkworm cocoon). (**e**) Response time of the pressure sensor (left: response time; right: recovery time). (**f**) Cycling durability of the flexible CEP-50 pressure sensor at pressure of 60 kPa.

As shown in Figure 5a, all current–voltage (I–V) curves demonstrate excellent linearity, indicating that the flexible CEP pressure sensor features a stable linear response behavior for constant pressure. As pressure grows, the I–V curve slope significantly rises since the conductivity of flexible CEP pressure sensors improves. Figure 5b exhibits the response curve of the flexible CEP sensor to a series of pressures from 3 kPa to 100 kPa at a constant frequency (0.01 Hz). The relative current change varies with the increasing loading pressure, and these response values are coincident with the results shown in Figure 4b. Figure 5c shows the responses of the flexible CEP sensor under cyclic loading at 120 kPa and frequencies ranging from 0.005 to 0.04 Hz. Similar response waveforms and peak amplitudes were observed within this tested range, indicating a consistent response under low-frequency cyclic loading. In addition, a small piece of silkworm (34 mg) was placed on the flexible CEP-50 sensor (15 mm × 15 mm), producing a detectable pressure of 1.5 Pa. This low detection limit implies that the CEP sensor is capable of detecting subtle signals, such as heartbeats and sound. Furthermore, the pressure sensor in Figure 5e shows a quicker response time (60 ms) and recovery time (60 ms). In Figure 5f, the current variation in the flexible CEP pressure exhibited negligible variations during 5000 loading–unloading cycles at a 60 kPa pressure with 0.01 Hz frequency. As shown in the amplified insets, current change waveforms are almost identical after each loading–unloading cycle, implying the reliable and stable dynamic durability of the pressure sensor.

Besides pressure, our flexible CEP pressure sensor has superior recognition of bending deformations. As shown in Figure S6a, bending sensing performance was evaluated. An arc with a length of 90 mm was repeatedly bent to verify the sensing performance of the as-fabricated CEP sensor (Figure S6b). The response curve of the flexible CEP pressure sensor to various bending deformations is shown in Figure S6c. Within the wide bending scope of a chord length from 89.9 to 85 mm, our CEP sensor can accurately monitor all bending deformations. In summary, the embossing patterns endow a flexible pressure sensor with superior sensing performance. Compared to the recently reported paper-based flexible pressure sensor, the obtained flexible CEP pressure sensor still shows excellent overall sensing performance.

### 3.3. Sensing Mechanism

**Figure 6:**
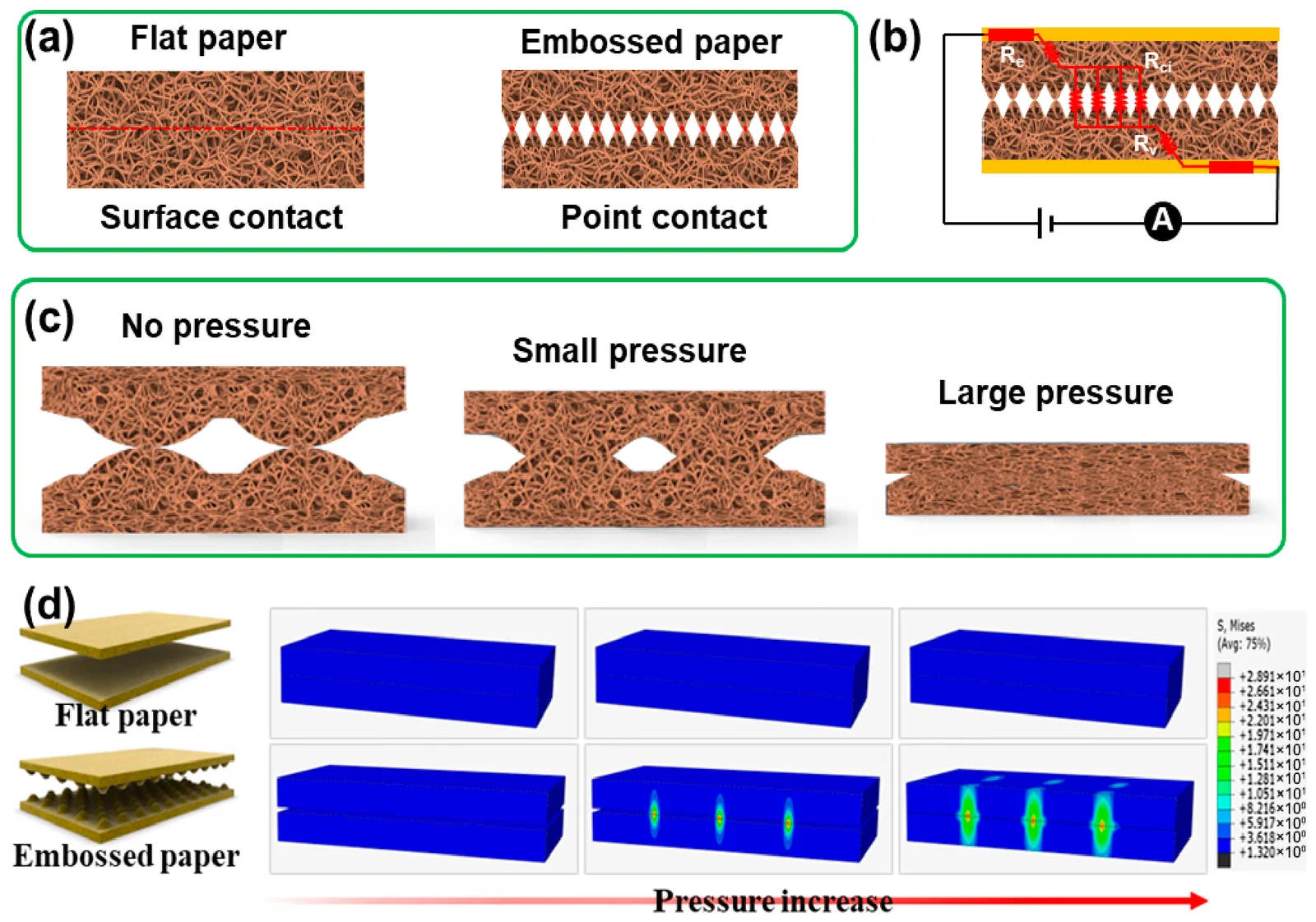
Electromechanical mechanism of the flexible CEP pressure sensor. (**a**) Cross-sectional model of two sheets of paper assembled face-to-face. (**b**) The equivalent circuit diagram of the flexible CEP pressure sensor. (**c**) Evolution models of a 3D micro-bump pattern under disparate pressures. (**d**) Equivalent stress distribution of flat paper and embossed paper under different pressures.

The piezoresistive response of the CEP sensor is associated with pressure-induced changes in the contact between the embossed surfaces and the porous carbonized fiber networks. As shown in Figure 5a, the approximately linear I–V curves exhibit increasing slopes with increasing pressure, indicating a decrease in the overall resistance. Figure 6a illustrates the arrangement of two CEP sheets with their embossed surfaces facing each other. Compared with flat sheets, the surface micro-bumps provide localized contact regions whose configuration may change under compression. Deformation of these micro-bumps may enlarge existing contacts and establish additional conductive pathways between the two sheets, contributing to the electrical response, particularly at low pressures. In addition to the surface microstructures, the porous fibrous network within each CEP sheet provides space for compression and rearrangement of internal fiber contacts, which may also affect the resistance. Our previous study on an electrospun fiber-based pressure sensor discussed how changes in fiber contact configurations under loading can influence sensing performance [10]. This provides a related structural perspective for interpreting the CEP response.

As illustrated in Figure 6b, the total resistance is represented by the simplified equivalent circuit:

R_t_ = R_e_ + R_c_ + 2R_v_

where R_t_ denotes total resistance; R_e_ denotes the combined electrode resistance; R_c_ represents the effective contact resistance between the two CEP sheets; and R_v_ denotes the effective bulk resistance of each sheet. During loading, R_e_ is treated as constant, and the resistance at the electrode–CEP interfaces is not considered separately.

As shown in Figure 6c, the pressure response of the CEP sensor can be interpreted through changes in the contact between the two embossed sheets and within their porous fiber networks. Before pressure is applied, the opposing micro-bumps form localized contacts, and voids remain within the carbonized fiber networks. Under a low applied pressure, the micro-bumps deform, allowing existing contact regions to expand and additional contacts to form between the two sheets. These changes may create more conductive paths across the interface and reduce the contact resistance, Rc, contributing to the high sensitivity observed at low pressures. Compression may also bring carbonized fibers within each sheet into closer contact, changing the internal conductive network and the bulk resistance, Rv. Both interfacial and internal contact changes may therefore contribute to the decrease in total resistance and the increase in current at a constant applied voltage. As pressure increases, the micro-bumps and porous fiber networks undergo further compression. The remaining space for deformation may become smaller, so that each additional pressure increment produces fewer changes in the contact arrangement and conductive pathways. This interpretation is consistent with the decreasing slopes of the current–pressure curve in Figure 4b.

To examine the effect of the embossed geometry on the mechanical behavior of the CEP structure, finite element analysis was performed to compare the equivalent stress distributions in flat and embossed paper structures under the prescribed compression conditions (Figure 6d). For the flat structure, stress was distributed relatively evenly across the contact region, indicating that the compressive load was carried over a broad area. In contrast, the embossed structure exhibited localized stress concentrations around the micro-bumps, with higher stresses at these locations than in the surrounding regions. This distribution reflects the nonuniform transmission of the load through the patterned interface. The micro-bumps act as localized load-bearing regions, producing a different stress distribution from that of the flat structure. The difference in stress distribution illustrates that embossed paper, compared to flat paper, is more likely to produce obvious structural deformations and greater contact area changes when subjected to pressure, leading to a larger change in the current.

### 3.4. Application of Flexible CEP-50 Pressure Sensor

**Figure 7:**
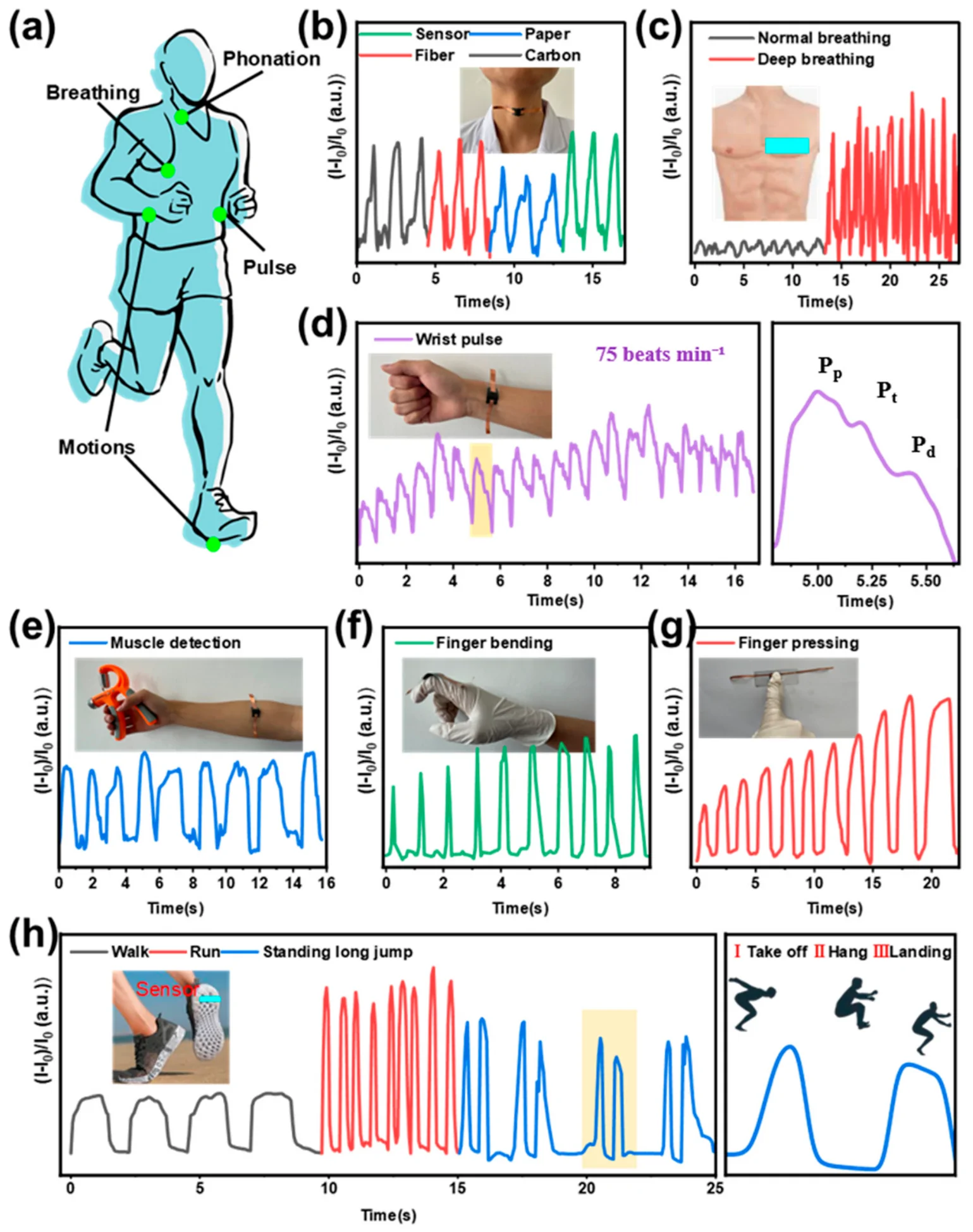
Application of flexible CEP pressure sensor. (**a**) Schematic representation of flexible CEP sensor used as a wearable device to monitor omni-directional human motions. (**b**) Real-time response of flexible CEP sensor to phonation (expressing disparate words like “Sensor”, “Fiber”, “Paper”, “Carbon”). (**c**) Real-time response of flexible CEP sensor for breathing. (**d**) Real-time response of flexible CEP sensor for pulse waveform. (The right image shows characteristic peaks of individual pulse waveforms). (**e**) Real-time response of flexible CEP sensor to muscle states. (**f**) Real-time response of flexible CEP sensor for finger bending. (**g**) Real-time response of flexible CEP sensor to finger pressure. (**h**) Real-time response of flexible CEP sensor for disparate motion states, including walking, running, and standing long jump. (The right image shows the whole process of the standing jump).

Profiting from its outstanding sensing performance, our flexible CEP sensor may serve as a wearable electronic device integrated into the human body to recognize omni-directional human activity. In Figure 7a, our flexible CEP pressure sensor was fixed to disparate body sites to monitor corresponding physiological or motion signals. Figure 7b shows recognition of the flexible CEP sensor for pronunciations. When volunteers articulated disparate words like “carbon”, “fiber”, “paper”, and “sensor”, the real-time signal curves tracked by the sensor also presented distinct waveforms. Figure 7c shows that current variations in the sensor resulted from “normal breathing” and “deep breathing”. It clearly shows two disparate physiological signals, “normal breathing” and “deep breathing”, which implies the possible application of our flexible CEP sensor in tracking respiratory diseases. Pulse is a critical physiological signal for diagnosing a patient’s disease category. As shown in Figure 7d, the flexible CEP sensor was used to monitor the volunteer’s radial artery pulse at the wrist in real time. The measured pulse rate was 75 beats min^−1^, which is within the normal resting range for healthy adults. The enlarged waveform on the right shows three characteristic components of the arterial pressure pulse: the percussion peak (P_p_), tidal peak (P_t_), and diastolic peak (P_d_) [52].

In addition to recognizing tiny physiological signals, vigorous human motions like joint motion, bending of a finger, and motion states can be detected by our flexible pressure sensor. As shown in Figure 7e, the CEP sensor was attached to the volunteer’s arm to monitor the state of the muscle. During exercise, the relaxation and stretching of muscles could be accurately distinguished. Figure 7f shows a flexible CEP pressure sensor attached to the finger joint to monitor finger bending. Figure 7g shows a CEP pressure sensor used as a pressure recognizer. It is clear that the value of the current change becomes more intense as the finger pressure increases. Figure 7h illustrates the attachment of a CEP pressure sensor to a shoe sole, which serves as a gait monitor for the recognition of various motion states, including walking, running, and standing long jump. As for the different motions, the curve of the current change demonstrates different response intensities and frequencies. The walking curve shows a typical moderate waveform with a cadence of 26 min^−1^, while the running curve exhibits a clear sharp waveform with a cadence up to 110 min^−1^. For the jumping state, the responsive curve shows a unique double-peaked waveform. The right-side image illustrates the whole process of the standing jump: phase I represents the preparation for take-off; phase II represents after jumping; and phase III represents the landing. This demonstration exhibits the remarkable benefits of our flexible CEP pressure sensor in effectively tracking omni-directional human motions.

## 4. Conclusions

In conclusion, an embossing pattern concept was adopted to fabricate a paper-based flexible pressure sensor through a three-step process consisting of embossing, carbonization, and assembly. During the embossing process, the metal mesh with a different mesh number was used as an embossed mold, which endowed the surface of pristine paper with a uniform array of micro-bump patterns. The results revealed that the density and geometry of embossing patterns are two essential factors affecting the sensing performance of the flexible CEP pressure sensor. Benefiting from the combination of a 3D fibrous network and micro-bump pattern structure, the as-obtained flexible CEP sensor exhibited superior sensing capabilities including high sensitivity (70.14 kPa^−1^), a wide working range (0–150 kPa), low detection limit (1.5 Pa), quick response time (60 ms), and excellent cycling durability (5000 cycles). Furthermore, an equivalent circuit and FEA were used to analyze the sensing mechanism behind the embossing pattern. Additionally, this flexible pressure sensor may serve as a monitor to detect various body movements, including physiological or motion signals. The clever utilization of a commercial paper-making technology to construct rational structures provides new insights into the development of flexible pressure sensors.

## Data Availability

The data presented in this study are available on request from the corresponding author.

## Supplementary Materials

The following supporting information can be downloaded at https://www.mdpi.com/article/10.3390/molecules31203736/s1. Figure S1 SEM images of embossing mould with different mesh number. (a) 30M; (b) 50M; (c) 70M; (d) 100M. Figure S2 Optical images of CEP sample before and after carbonization. (a) The optical image of pristine embossed paper before carbonization. (b) The optical image of CEP sample. (c) The optical image of flexible CEP sample. Figure S3 Full XPS spectrum of embossed paper before and after carbonization. Figure S4. High-resolution C 1s XPS spectra and peak deconvolution of embossed cellulose paper (a) before and (b) after carbonization. Figure S5 Raman spectrum of embossed paper before and after carbonization. Figure S6 The sensing performance of flexible pressure sensor for bending deformations. (a) the image of an own-house built test device for the evaluation of bending sensing performance of the sensor. (b) Schematic diagram of the shape of the sensor during bending. (c) Relative current changes of the pressure sensor at different bending degrees. Table S1. The comparison of the sensing performance of paper based flexible pressure sensor reported in the recent literatures.
