# Supplementary material for: Embossing-Controlled Hierarchical Structures in Cellulose-Derived Carbon Paper for Flexible Piezoresistive Pressure Sensing
Source: Molecules. 2026 Oct 18;31(20):3736. doi: 10.3390/molecules31203736 (PMC13707585; doi:10.3390/molecules31203736)
Supplement: Supplementary file 1 [file molecules-31-03736-s001.zip › molecules-4600796-supplementary.pdf]

Supporting Information

Embossing-controlled Hierarchical Structures in Cellulose-derived Carbon Paper for Flexible Piezoresistive Pressure Sensing

Wei Wang[1,2,3,4*], Luyao Chen[1], Zhixiang Wang[1], Youbo Di[1], Yaru Yang[2,*], Yunchao Xiao[2], Shuo Yang[3,4*], Hua Wang[1]

1 College of Textile Engineering, Taiyuan University of Technology, Taiyuan 030600, Shanxi, China

2 College of Materials and Textile Engineering, Jiaxing University, Jiaxing 314001, China

3 School of Light Industry Science and Engineering, Tianjin University of Science and Technology, Tianjin 300457, China

4 State Key Laboratory of Bio-based Fiber Materials, China Textile Academy, Beijing 100025, China

* Corresponding Authors: wangwei03@tyut.edu.cn(W. Wang); yyr0515@zjxu.edu.cn(Y. Yang); yangshuo1018@tust.edu.cn(S. Yang)

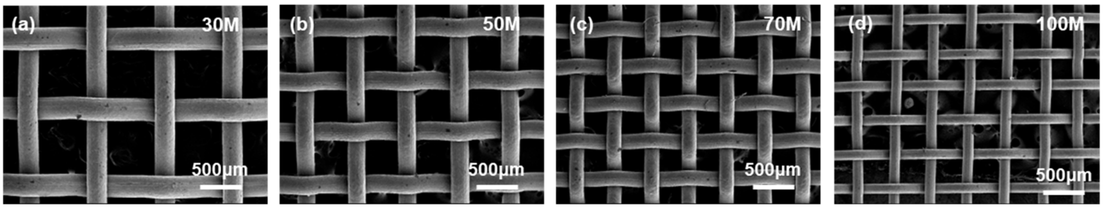

Figure S1 SEM images of embossing mould with different mesh number. (a) 30M; (b) 50M; (c) 70M; (d) 100M

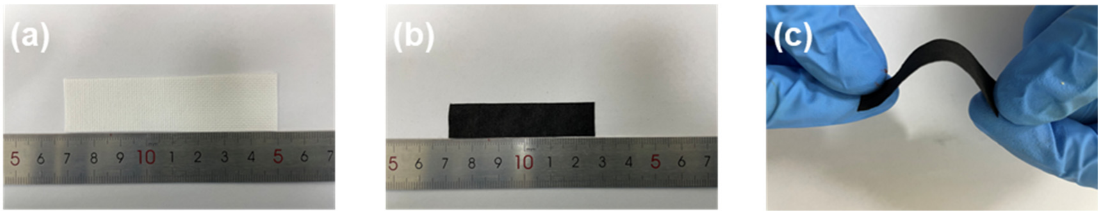

Figure S2 Optical images of CEP sample before and after carbonization. (a) The optical image of pristine embossed paper before carbonization. (b) The optical image of CEP sample. (c) The optical image of flexible CEP sample.

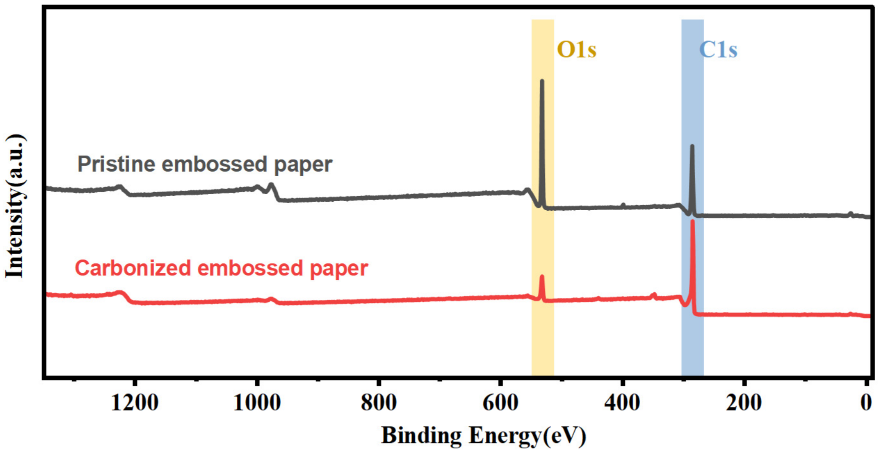

Figure S3 Full XPS spectrum of embossed paper before and after carbonization

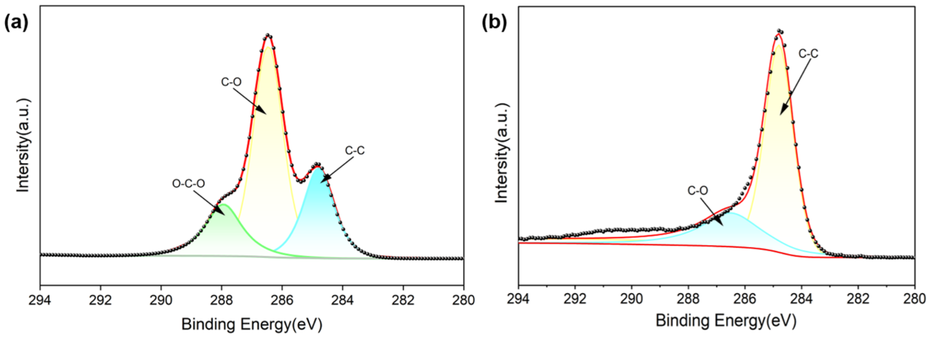

Figure S4. High-resolution C 1s XPS spectra and peak deconvolution of embossed cellulose paper (a) before and (b) after carbonization.

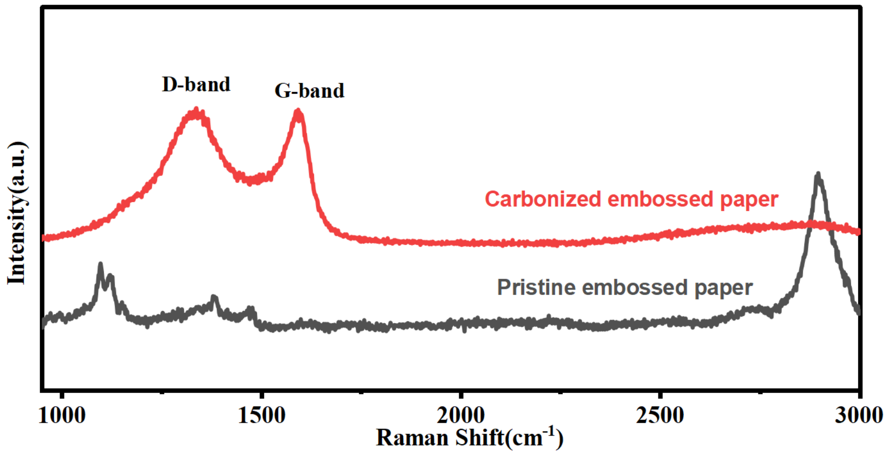

Figure S5 Raman spectrum of embossed paper before and after carbonization

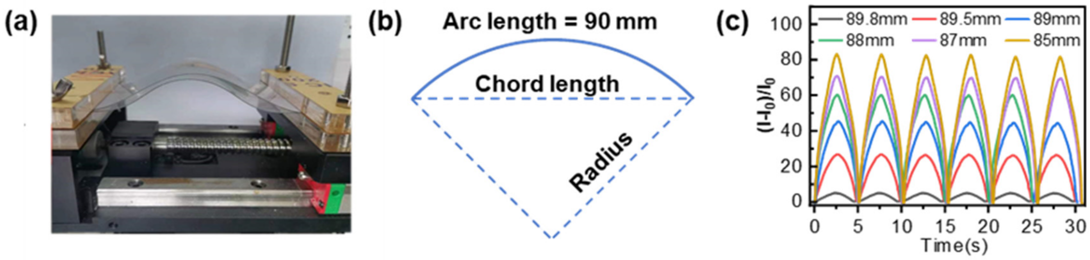

Figure S6 The sensing performance of flexible pressure sensor for bending deformations. (a) the image of an own-house built test device for the evaluation of bending sensing performance of the sensor. (b) Schematic diagram of the shape of the sensor during bending. (c) Relative current changes of the pressure sensor at different bending degrees.

Table S1. The comparison of the sensing performance of paper based flexible pressure sensor reported in the recent literatures.

Active materials	Sensitivity (kPa^{-1})	Working range (kPa)	Detection limit (Pa)	Response/recovery time (ms)	Dynamical stability	Ref.
Carbonized crepe paper	2.56-5.67(1-2.53kPa)<br>0.87(2.53-9.06kPa)<br>0.32(9.06-20kPa)	0-20	0.9	30	3000	[1]

Tissue paper with AgNWs	1.5(0.03-30.2kPa)	0.03-30.2	30	N/A	N/A	[5]
Air-laid paper with carbon black	51.23(0-10Pa)	0-30	1	200	10000	[32]
PPy tissue paper	4.8(0-5.5kPa)<br>1.7(5.5-40kPa)	0-40	N/A	N/A	N/A	[33]
Mxene-coated tissue paper	509.5(0-0.5kPa)<br>179.4(10-30kPa)<br>53.7(30-100kPa)	0-100	1	N/A	10000	[48]
Air-laid paper with rGO and Polyaniline	143.41(0-0.5kPa)<br>0.08(0.5-40kPa)	0-40	5	70/100	5000	[49]
WSe_2 nanosheets decorated tissue paper	29.24(1-12Pa)<br>11.94(2-30kPa)<br>3.2(35-100kPa)	1-100	1.4	70/100	N/A	[11]
Graphene-paper	17.2(0-2kPa)<br>0.1(2-20kPa)	0-20	N/A	120/60	300	[35]
Polyaniline decorated cellulose paper	2.23(5-22kPa)	2-90	N/A	70/100	800	[14]
SnS/paper	3.18(0.9-25kPa)	0.9-25	N/A	300	1000	[39]
Mxene-impregnated tissue paper	0.55(23-982Pa)<br>3.81(0.982-10kPa)<br>2.52(10-30kPa)	0-30	10.2	11	10000	[47]
Gold nanowires impregnated tissue paper	1.14(0-5kPa)	0-5	13	17	50000	[29]
Paper/carbon ink	0.614(0-6kPa)<br>0.0064(6-50kPa)	0-50	N/A	235	5000	[50]
Paper/carbon nanotube	2.2(0.35-2.5kPa)<br>1.3(2.5-11.7kPa)	0-11.7	35	35-40	N/A	[51]
Mxene/BC film	51.14(0-0.82kPa)<br>2.62(0.82-10.92kPa)	0-10.92	N/A	99/93	5000	[52]
Carbonized embossed paper	70.14(0-0.2kPa)<br>1.74(0.2-35kPa)<br>0.66(35-90kPa)<br>0.28(90-150kPa)	0-150	1.5	60/60	5000	This work
